# Nano-electrospray tandem mass spectrometric analysis of the acetylation state of histones H3 and H4 in stationary phase in *Saccharomyces cerevisiae*

**DOI:** 10.1186/1471-2091-12-34

**Published:** 2011-07-04

**Authors:** Mzwanele Ngubo, Gabré Kemp, Hugh G Patterton

**Affiliations:** 1 Department of Biotechnology, University of the Free State, Bloemfontein, South Africa; 2 Facility for Genomics and Proteomics, University of the Free State, Bloemfontein, South Africa; 3 Advanced Biomolecular Research Cluster, University of the Free State, Bloemfontein, South Africa

## Abstract

**Background:**

The involvement of histone acetylation in facilitating gene expression is well-established, particularly in the case of histones H3 and H4. It was previously shown in *Saccharomyces cerevisiae *that gene expression was significantly down-regulated and chromatin more condensed in stationary phase compared to exponential phase. We were therefore interested in establishing the acetylation state of histone H3 and H4 in stationary and in exponential phase, since the regulation of this modification could contribute to transcriptional shut-down and chromatin compaction during semi-quiescence.

**Results:**

We made use of nano-spray tandem mass spectrometry to perform a precursor ion scan to detect an *m/z *126 immonium ion, diagnostic of an N^ε^-acetylated lysine residue that allowed unambiguous identification of acetylated as opposed to tri-methylated lysine. The fragmentation spectra of peptides thus identified were searched with Mascot against the Swiss-Prot database, and the y-ion and b-ion fragmentation series subsequently analyzed for mass shifts compatible with acetylated lysine residues. We found that K9, K14 and K36 of histone H3 and K12 and K16 of histone H4 were acetylated in exponential phase (bulk histones), but could not detect these modifications in histones isolated from stationary phase cells at the sensitivity level of the mass spectrometer. The corresponding un-acetylated peptides were, however, observed. A significantly higher level of acetylation of these residues in exponential phase was confirmed by immuno-blotting.

**Conclusion:**

H4K16 acetylation was previously shown to disrupt formation of condensed chromatin *in vitro*. We propose that de-acetylation of H4K16 allowed formation of condensed chromatin in stationary phase, and that acetylation of H3K9, H3K14, H3K36, and H4K12 reflected the active transcriptional state of the yeast genome in exponential phase.

## Background

In 1963 Allfrey and colleagues showed that the acetylation of histone H3 and H4 alleviated the repressive effect of the histones on *in vitro *RNA synthesis in calf nuclei [1,2]. This initial report saw the birth of epigenetics and an appreciation that covalent modifications of histones provided a mechanism whereby DNA function could be regulated. The nucleosomal packaging of DNA in chromatin was subsequently shown to be an integral component in the regulatory mechanism of the transcription process [3], involving ATP-dependent remodeling complexes, reversible, chemical modification of core histone as well as histone isotype swapping. Many transcriptional activators and repressors were further shown to be enzymes that were involved in the covalent modification of function-specific residues in the core histones, mostly located in the N- and C-terminal "tails" that extend from the central histone-fold domain [4].

It is now known that these chemical modifications are crucial to the proper regulation of DNA function. The absence of many of the proteins involved in post-translational histone modifications underlie many human diseases, including T-cell lymphoma, Rubinstein-Taybi syndrome, Coffin-Lowry syndrome and the autism spectrum disorders [5]. In addition, the proper regulation of histone modifying enzymes are required for normal muscle development, neuronal plasticity as well as memory functions [6].

A general relationship between acetylation of the core histones and transcriptional activity, combined with chromatin decondensation, has been reported by many different groups [7]. We have previously shown that linker histone Hho1 displayed a significant increase in chromatin binding in stationary phase in *Saccharomyces cerevisiae*, which coincided with an increase in chromatin compaction [8]. Gene expression and protein synthesis were significantly decreased in stationary phase in *S. cerevisiae*, allowing cells to survive for extended periods in this semi-quiescent state [8-10]. Following re-feeding of the stationary phase yeast cells, the linker histone rapidly dissociated from chromatin, accompanied by a general increase in transcriptional activity as cells re-entered the cell cycle [8]. We were therefore interested in investigating the presence of acetyl groups on all the N-terminal tail lysine residues of H3 and H4 in stationary and in exponential phase in *S. cerevisiae*, to elucidate the role that acetylation may play in transcriptional shut-down and compaction of the chromatin fiber during semi-quiescence.

## Methods

### Strains and culture media

*Saccharomyces cerevisiae *strain W303-1A (*MAT***a**, *leu2-3, 112trp1-1, can1-100, ura3-1, ade2-1, his3-11,15*) was grown in YPD medium (1% w/v yeast extract, 2% w/v bacto-peptone, 2% w/v glucose) at 30°C with constant shaking to exponential phase (approximately 10^7 ^cells/ml) or to stationary phase (6 day culture). Yeast cells were pelleted, spheroplasted with zymolyase, nuclei isolated, and core histones extracted as described [11]. The procedure was performed at 4°C in the presence of the HDAC inhibitor sodium butyrate to minimize changes in the acetylation level of histones during isolation. Histone integrity was verified by SDS-PAGE electrophoresis followed by staining with Coomassie Brilliant Blue [11].

### In-gel digestion

A gel slice containing the Coomassie-stained histone band of interest was excised and cut into small cubes (~1 mm^3^). The gel pieces were shrunk with 500 μl acetonitrile (Sigma). A 50 μl aliquot of 10 mM dithiothreitol in 100 mM NH_4_HCO_3 _(Sigma) was added to cover the gel pieces completely, and the sample incubated at 56°C for 30 min. The sample was cooled to room temperature, 50 μl of 55 mM iodoacetamide (Sigma) in 100 mM NH_4_HCO_3 _was added, and the sample incubated in the dark at room temperature for 20 min. Following reductive alkylation, Coomassie-stained gel pieces were destained for 30 min in 100 μl of 100 mM NH_4_HCO_3_/acetonitrile (1:1, v/v) with occasional vortexing. The gel pieces were saturated with sequencing grade trypsin (Promega) (10 μg/ml in 10 mM NH_4_HCO_3_, 10% v/v acetonitrile), incubated on ice for 90 min, followed by overnight incubation at 37°C. Peptides were extracted from the gel matrix in 100 μl of 5% v/v formic acid (Merck), 60% v/v acetonitrile at 30°C for 30 min with gentle shaking. The recovered supernatant was dried down in a rotary evaporator (Savant) and the peptides resuspended in 20 μl of 5% v/v formic acid for MS analysis.

### Mass spectrometric analysis

Samples (5 μl) were concentrated and desalted online on a 5 mm × 300 μm Zorbax 300SB-C18 trapping column (Agilent) and the peptides separated on a 150 mm × 75 μm Zorbax 300SB-C18 column with 3.5 μm particle size by nanoflow HPLC (Agilent model 1200). The following step-wise gradient profile was used at a flow-rate of 0.35 μl/min: 10 min, 0% B; 15 min, 10% B; 95 min, 25% B; 100 min 50% B; 101 min, 90% B; 120 min 90% B. Solvent A consisted of 1% v/v acetonitrile, 0.1% v/v formic acid and Solvent B consisted of 95% v/v acetonitrile, 0.1% v/v formic acid. The separated peptides were analyzed on a 4000 QTRAP hybrid triple quadrupole mass spectrometer (AB SCIEX) fitted with a nano electrospray-ionisation interface, operated in "Information Dependent Acquisition" (IDA) mode using Analyst version 1.5 software (AB SCIEX). A precursor ions scan between *m/z *400-1400 was performed as a survey scan for the IDA method. Peptide precursors that generated an *m/z *126 fragment during collision induced dissociation (CID) were identified, and peaks above 1000 counts/s triggered the IDA method to perform an enhanced resolution scan for charge state determination, followed by enhanced product ion (EPI) scan of the precursor at rolling collision energy settings.

#### Protein identification and modification discovery by database search

The recorded MS/MS spectra were submitted to a local server running Mascot version 2.1 (Matrix Science) and searched against the Swiss-Prot (version 51.6) database. Precursor and product ion tolerances were 1.2 and 0.6 Da, respectively, appropriate for the accuracy of the 4000 QTRAP. Acetylation of lysine was specified as a variable modification and up to two missed cleavages by trypsin were allowed.

### Western blots

Core histones, isolated from exponential and stationary phase yeast cells, were resolved by SDS-PAGE, transferred to Hybond-ECL membrane (GE Healthcare), and the immobilized proteins probed with polyclonal rabbit antibodies targeted at H3 K9Ac, H3 K14Ac, H4 K12Ac and H4 K16Ac (Abcam; AB10812, AB46984, AB46983 and AB61240 respectively). Antibody binding was visualized with an ECL Western Blot kit (GE Healthcare) according to the manufacturer's instructions, and the image digitized with a Pharos Molecular Imager (Bio-Rad).

## Results

We set out to identify the residues that were acetylated in core histones of *Saccharomyces cerevisiae *in stationary and exponential phase to establish a possible role for histone acetylation during semi-quiescence. Histone H4 was isolated from exponential phase *S. cerevisiae *cells, resolved by SDS-PAGE, the H4 containing gel slice excised, the histone digested with trypsin in the gel slice, and the resulting peptides eluted and subjected to MS analysis.

It was previously shown that the immonium ion of acetylated lysine often lost an ammonia molecule, resulting in the immonium ion sub-fragment with a mono-isotopic mass of 126.0919 Da [12]. This immonium ion sub-fragment was previously used as a diagnostic ion to detect acetylated lysine residues in peptides during CID fragmentation [12]. This allowed unambiguous assignment of an acetylated lysine residue, since trimethylated lysine exhibits a similar mass shift in the fragmentation spectrum. We therefore performed precursor ion scans during elution to identify all peptides that generated an *m/z *126 ion during CID fragmentation. These peptides were selected when present in a peak with a height exceeding a total ion count (TIC) of 1000 counts/second, and a product ion scan of each recorded. All product ion scans were submitted to the Mascot search engine, which matched the experimental MS/MS spectra against the generated theoretical spectra of all yeast proteins in the Swiss-Prot database.

**Figure 1:**
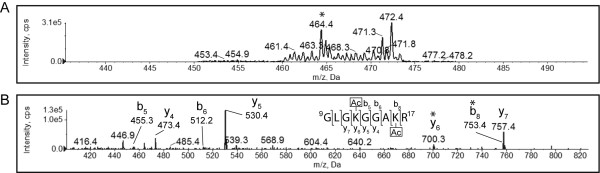
**K12 and K16 of histone H4 are acetylated in exponential phase**. A tryptic digest of histone H4 isolated from exponential phase yeast cells was analyzed by MS. The enhanced precursor ion spectrum (A) and CID fragmentation spectrum of the peptide that resolved at *m/z *464.4 ([M+2H]^2+^), indicated by the asterisk in panel A, is shown (B). The partial y-ion and b-ion series visible in the fragmentation spectrum are indicated, and the y_6 _and b_8 _peaks, representing terminal lysine residues shifted by an additional 42 Da, indicated by asterisks. The full *m/z *list of observed and predicted fragments are given in Table 1.

Of all the peptides generated by tryptic cleavage of H4 that generated peaks with a TIC of at least 1000 counts/second, only the peptide from position 9-17 produced a diagnostic ion at *m/z *126, indicating the presence of at least one acetylated lysine residue in the peptide. A di-acetylated H4 fragment was identified when performing a Mascot search with the fragmentation spectrum of this peptide against the Swiss-Prot database. This assignment was supported by the precursor ion of *m/z *464.3723 for [M+2H]^2+ ^(see Figure 1A), recorded by the enhanced resolution scan of the ion. This is within approximately 0.1 Da of the expected mass of the peptide GLGKGGAKR with *m/z *843.5159 for the [M+H]^+ ^ion and 42.0106 Da for each of the acetyl groups. This result provided very strong evidence that the peptide fragment from 9-17 of H4 was di-acetylated. Since this fragment contains only two lysine residues, it is very likely that both K12 and K16 were acetylated. To confirm this assignment, the CID fragmentation spectrum was also considered. Partial b- and y-ion fragmentation series could be identified (see Figure 1B). Looking first at the y-ion series, the y_4_, y_5_, y_6 _and y_7 _fragments were visible. Lysine 12 will thus correspond to the N-terminal residue in the y_6 _fragment. The mass difference between the y_5 _and y_6 _fragment was 170.1055 Da (see Table 1). This was within 0.1 Da of the mass of an acetylated lysine residue, which is equal to 128.09 Da plus 42.0106 Da. This result demonstrated that lysine 12 of H4 was acetylated in exponential phase *S. cerevisiae *cells.

**Table 1:** Fragment ions of the ^9^GLGKGGAKR^17 ^peptide from histone H4 isolated from exponential phase *Saccharomyces cerevisiae*.

Order	b ion (*m/z*)	Sequence	y ion (*m/z*)	Order
1	58.0287	G		9
2	171.1128	L	870.5156	8
3	228.1343	G	757.4315*	7
4	398.2398	K	700.4100*	6
5	455.2613*	G	530.3045*	5
6	512.2827*	G	473.2831*	4
7	583.3198	A	416.2616*	3
8	753.4254*	K	345.2245	2
9		R	175.1190	1

The list of product ions observed (underlined), matched with Mascot to the SWISS-PROT database (asterisk superscript), or predicted following CID fragmentation is shown.

Looking next at the b-ion series (Figure 1B), the b_5_, b_6 _and b_8 _ions could be identified. K16 would be the C-terminal residue in the b_8 _fragment. Since the b_7 _fragment was not observed, possibly due to the lower proton affinity of this fragment, it was not possible to directly calculate the mass of the C-terminal residue in b_8_. However, the difference in mass of the b_6 _(512.2827 Da) and b_8 _(753.4254 Da) fragments was 241.1427 Da. The mass of the di-peptide AK with the lysine acetylated is 241.13, within 0.1 Da of the observed mass. This provided very strong evidence that the second acetyl group was present on K16 of the H4 fragment.

To investigate the acetylation state of histone H4 in semi-quiescent *S. cerevisiae*, we isolated H4 from 6 day stationary phase yeast cells, performed a tryptic digest of the sample resolved on an SDS-PAGE gel, and analyzed the eluted peptide fragments by MS. Since a precursor ion scan for a diagnostic ion was used, a peptide fragment that was acetylated should still be detected, even if additional post-translational modifications that could influence the chromatographic elution property of the peptide were present in stationary phase.

**Figure 2:**
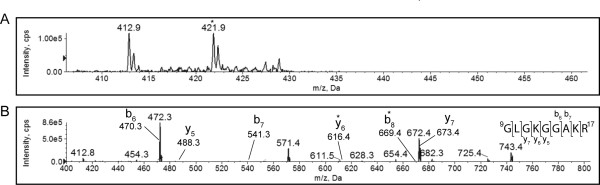
**K12 and K16 of histone H4 are not detectably acetylated in stationary phase**. A tryptic digest of histone H4 isolated from stationary phase yeast cells was analyzed by MS. The enhanced precursor ion spectrum (A) and CID fragmentation spectrum of the peptide that resolved at *m/z *421.9 ([M+2H]^2+^), indicated by the asterisk in panel A, is shown (B). The partial y-ion and b-ion series visible in the fragmentation spectrum are indicated, and the y_6 _and b_8 _peaks, representing terminal lysine residues, indicated by asterisks. The full *m/z *list of observed and predicted fragments are given in Table 2.

We could not detect the diagnostic ion at *m/z *126 during a precursor ion scan in any of the peptides generated by tryptic cleavage of H4. This result suggested that H4 was either not acetylated in yeast in stationary phase, or acetylated at a significantly reduced level, below our selected detection cut-off of 1000 counts/second. However, since ion-suppression in different samples may influence the generation of the immonium ion sub-fragment, we also studied the fragmentation spectra of the peptides (Figure 2).

A precursor peak at *m/z *421.9 was visible for the ^9^GLGKGGAKR^17 ^peptide from stationary phase H4 (Figure 2A). The mass at which this peak resolved was consistent (within 0.4 Da) with the unmodified, doubly charged peptide GLGKGGAKR. Looking next at the MS/MS spectrum, partial b-ion and y-ion series were visible (Figure 2B). The mass difference between y_5 _and y_6_, where K12 is present as the N-terminal residue, was 128.0949 Da (see Table 2), within 0.1 Da of the mono-isotopic mass of unmodified lysine. Lysine 16 is the C-terminal residue of the b_8 _ion. The mass difference between b_7 _and b_8 _was 128.095 Da, also within 0.1 Da of the mass of an unacetylated lysine residue. This result demonstrated that, in contrast to exponential phase, both lysine 12 and 16 of histone H4 were not acetylated or acetylated at significantly reduced levels in stationary phase. This reduced level of histone acetylation was not due to the action of deacetylases during the histone isolation procedure, since we included the deacetylase inhibitor butyrate, and histones isolated from exponential phase using the identical procedure displayed high levels of acetylation, consistent with inactive deacetylases.

**Table 2:** Fragment ions of the ^9^GLGKGGAKR^17 ^peptide from histone H4 isolated from stationary phase *Saccharomyces cerevisiae*.

Order	b ion (*m/z*)	Sequence	y ion (*m/z*)	Order
1	58.0295	G		9
2	171.1135	L	786.5192	8
3	228.1350	G	673.4351	7
4	356.2299	K	616.4136	6
5	413.2514	G	488.3187	5
6	470.2729	G	431.2972	4
7	541.3100	A	374.2757	3
8	669.4050	K	303.2386	2
9		R	175.1437	1

The list of product ions observed (underlined), matched with Mascot to the SWISS-PROT database (asterisk superscript), or predicted following CID fragmentation is shown.

**Figure 3:**
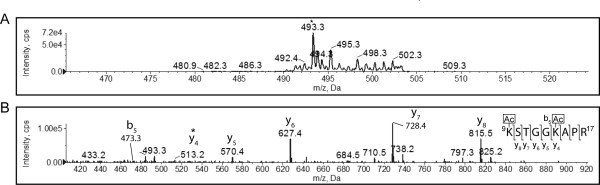
**K9 and K14 of histone H3 are acetylated in exponential phase**. A tryptic digest of histone H3 isolated from exponential phase yeast cells was analyzed by MS. The enhanced precursor ion spectrum (A) and CID fragmentation spectrum of the peptide that resolved at *m/z *493.3, indicated by the asterisk in panel A, is shown (B). The partial y-ion and b-ion series visible in the fragmentation spectrum are indicated, and the y_4 _peak, shifted by 42 Da relative to the predicted y_3 _*m/z*, is indicated by an asterisk. The full *m/z *list of observed and predicted fragments are given in Table 3.

We next asked whether the acetylation state of histone H3 also differed between exponential and stationary phase in yeast. Histone H3 was isolated from exponential and stationary phase yeast cells, resolved by SDS-PAGE, the histone gel band excised, and digested with trypsin. The digest was analyzed by MS as described above using a precursor ion scan in an IDA method. We detected two acetylated peptides in histone H3 isolated from exponential phase, based on the presence of the *m/z *126 fragment ion. A Mascot search of the Swiss-Prot database with the MS/MS spectrum identified the first peptide as ^9^KSTGGKAPR^17 ^of H3. In its un-acetylated form this peptide has a predicted mass of 451.26 Da in its doubly charged state. The enhanced resolution scan of this peptide exhibited a mass of 493.3 for [M+2H]^2+ ^(Figure 3A), a difference of 42 Da, consistent with the presence of two acetyl groups on this peptide. Looking at the fragmentation spectrum (Figure 3B) partial y-ion and b-ion series were visible. It was not possible to directly view the N-terminal lysine residue, since the b_1 _ion resolved below the *m/z *scan range. However, the y_8 _ion was detected at *m/z *815.437. Thus the mass difference between the full peptide (985.6 Da for the singly charged state) and the y_8 _ion was 170.163 Da, which was most consistent with a single acetylated lysine residue. Since neither the y_3 _or b_6 _ions were observed in the fragmentation spectrum, it was not possible to directly calculate the mass of K14. However, the mass predicted for the y_3 _ion of the KSTGGKAPR peptide was 343.2088 Da (see Table 3). The y_4 _ion was observed at *m/z *513.3144. Thus, the N-terminal residue of the y_4 _fragment had a mass of 170.1056 Da, consistent with an acetylated lysine residue. Furthermore, the mass difference between the observed b_6 _and predicted b_5 _ions was 170.1056 Da, again consistent with an acetylated lysine residue. We therefore conclude that both K9 and K14 were acetylated in histone H3 in exponential phase yeast cells.

**Table 3:** Fragment ions of the ^9^KSTGGKAPR^17 ^peptide from histone H3 isolated from exponential phase *Saccharomyces cerevisiae*.

Order	b ion (*m/z*)	Sequence	y ion (*m/z*)	Order
1	171.1128	K		9
2	258.1448	S	815.4370*	8
3	359.1925	T	728.4050*	7
4	416.2140	G	627.3573*	6
5	473.2354	G	570.3358*	5
6	643.3410	K	513.3144*	4
7	714.3781	A	343.2088	3
8	811.4308	P	272.1717	2
9		R	175.1190	1

The list of product ions observed (underlined), matched with Mascot to the SWISS-PROT database (asterisk superscript), or predicted following CID fragmentation is shown.

**Figure 4:**
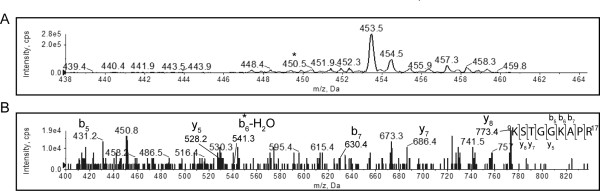
**K9 and K14 of histone H3 are not detectably acetylated in stationary phase**. A tryptic digest of histone H3 isolated from stationary phase yeast cells was analyzed by MS. The enhanced precursor ion spectrum (A) and CID fragmentation spectrum of the peptide that resolved at *m/z *450.5, indicated by the asterisk in panel A, is shown (B). The partial y-ion and b-ion series visible in the fragmentation spectrum are indicated, and the b_6_^0 ^ion, shifted by 42-18 Da relative to b_5_, is indicated by an asterisk. The full *m/z *list of observed and predicted fragments are given in Table 4.

To investigate whether these residues were also acetylated in stationary phase, histone H3 was isolated from 6 day stationary phase cells and analyzed as above. We could not detect the *m/z *126 ion fragment in the precursor-ion scan of the tryptic digest for peptides present at a level above our IDA cut-off of 1000 counts/second. We therefore performed an enhanced MS scan to identify the KSTGGKAPR peptide. An *m/z *450.5 peak was identified (Figure 4A), within 0.8 Da of the mass that was expected for the doubly charged peptide. Searching the fragmentation spectrum of the peptide that eluted in this peak against the Swiss-Prot database with Mascot, indeed identified the un-acetylated KSTGGKAPR peptide as the best match. Looking at the fragmentation spectrum of this peptide (Figure 4B) partial b-ion and y-ion series were visible. Again, the b_1 _ion, containing the N-terminal K9, was below the *m/z *range of the scan (Figure 4B). The mass difference between the full-length [M+H]^+ ^peptide (*m/z *901.5) and the y_8 _ion (*m/z *773.4264) was 128.0736 Da (see Table 4), within 0.1 Da of the mass of an un-acetylated lysine residue. Looking next at K14, a b_5 _(*m/z *431.2249) and a b_6_^0 ^(*m/z *541.3093) fragment were observed, the latter having lost a water molecule. Thus, the observed mass of K14 was 128.0844 Da, within 0.1 Da of the mass of an un-acetylated lysine residue. This result clearly indicated that both K9 and K14 were un-acetylated or acetylated at low levels in histone H3 isolated from stationary phase yeast cells.

**Table 4:** Fragment ions of the ^9^KSTGGKAPR^17 ^peptide from histone H3 isolated from stationary phase *Saccharomyces cerevisiae*.

Order	b ion (*m/z*)	b^0 ^ion (*m/z*)	Sequence	y ion (*m/z*)	y^0 ^ion (*m/z*)	Order
1	129.1022		K			9
2	216.1343	198.1237	S	773.4264*	755.4159	8
3	317.1819	299.1714	T	686.3944*	668.3838	7
4	374.2034	356.1928	G	585.3467		6
5	431.2249*	413.2143*	G	528.3253*		5
6	559.3198	541.3093*	K	471.3038		4
7	630.3570*	612.3464*	A	343.2088		3
8	727.4097*	709.3991	P	272.1717		2
9			R	175.1190		1

The list of product ions observed (underlined), matched with Mascot to the SWISS-PROT database (asterisk superscript), or predicted following CID fragmentation is shown.

**Figure 5:**
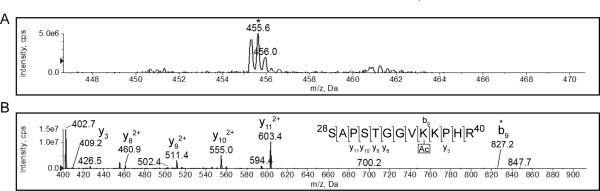
**Histone H3 K36, but not K37, is acetylated in exponential phase**. A tryptic digest of histone H3 isolated from exponential phase yeast cells was analyzed by MS. The enhanced product ion spectrum (A) and CID fragmentation spectrum of the peptide that resolved at 455.6 Da, indicated by the asterisk in panel A, is shown (B). The partial y-ion and b-ion series visible in the fragmentation spectrum are indicated, and the b9 peak, shifted by 42 Da relative to the mass of lysine, is indicated by an asterisk. The full *m/z *list of observed and predicted fragments are given in Table 5.

Apart from the ^9^KSTGGKAPR^17 ^H3 peptide, the precursor ion scan of the tryptic digest of H3 isolated from exponential phase also identified an *m/z *126 Da diagnostic ion in a *m/z *455.6 peptide (Figure 5A). A Mascot search of the Swiss-Prot database with the fragmentation spectrum of this peptide identified the sequence SAPSTGGVKKPHR, located at sequence position 28-40 of yeast H3. The predicted mass of this peptide was 1321.73 Da ([M+H]^+^), 42 Da less than the observed mass of 1363.74 Da (the observed mass of 455.3 Da is for the triply charged peptide). This 42 Da mass difference is consistent with the presence of a single acetyl group in the peptide, which may be located on either K36 or K37. In the fragmentation spectrum (Figure 5B) the b_9 _ion, containing K36 as the C-terminal residue, was observed at *m/z *827.4258 Da (see the asterisk in Figure 5B),. The predicted mass of b_8_, which contained residues that are not acetylatable, was 657.3202 Da (see Table 5). Thus, the difference between b_8 _and the observed b_9 _fragment was 170.1056 Da, which is within 0.1 Da of an acetylated lysine residue. Thus, the single acetyl group detected in the fragment was present on K36 of H3. K37 was not detectably acetylated.

**Table 5:** Fragment ions of the ^28^SAPSTGGVKKPHR^40 ^peptide from histone H3 isolated from exponential phase *Saccharomyces cerevisiae*.

Order	b ion (m/z)	b^2+ ^ion (m/z)	Sequence	y ion (m/z)	y^2+ ^ion (m/z)	Order
1	88.0393	44.5233	S			13
2	159.0764	80.0418	A	1276.7120	638.8597	12
3	256.1292	128.5682	P	1205.6749	603.3411*	11
4	343.1612	172.0842	S	1108.6222	554.8147*	10
5	444.2089	222.6081	T	1021.5901	511.2987*	9
6	501.2304	251.1188	G	920.5425	460.7749*	8
7	558.2518	279.6295	G	863.5210	432.2641	7
8	657.3202	329.1638	V	806.4995	403.7534*	6
9	827.4258	414.2165	K	707.4311	354.2192	5
10	955.5207	478.2640	K	537.3256*	269.1664	4
11	1052.5735	526.7904	P	409.2306*	205.1190	3
12	1189.6324	595.3198*	H	312.1779	156.5926	2
13			R	175.1190	88.0631	1

The list of product ions observed (underlined), matched with Mascot to the SWISS-PROT database (asterisk superscript), or predicted following CID fragmentation is shown.

**Figure 6:**
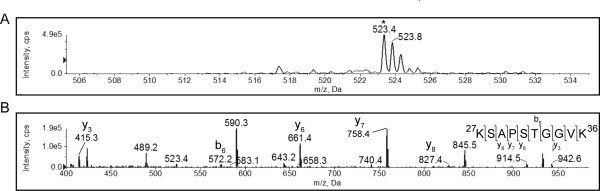
**Histone H3 K36 is not detectably acetylated in stationary phase**. A tryptic digest of histone H3 isolated from stationary phase yeast cells was analyzed by MS. The enhanced product ion spectrum (A) and CID fragmentation spectrum of the peptide that resolved at *m/z *523.4, indicated by the asterisk in panel A, is shown (B). The partial y-ion and b-ion series visible in the fragmentation spectrum are indicated. The full *m/z *list of observed and predicted fragments are given in Table 6.

We also identified the partially overlapping ^27^KSAPSTGGVK^36 ^peptide at *m/z *523.4 in the precursor ion scan on the tryptic digest of histone H3 isolated from stationary phase yeast cells (Figure 6A) using a Mascot search of the Swiss-Prot database. This fragment was never observed in conjunction with the *m/z *126 fragment ion. However, the predicted mass for the doubly charged peptide was 466.23 Da. Thus, in its singly charged state, the peak identified as KSAPSTGGVK was approximately 112 Da heavier than predicted. Nevertheless, the y-ion series, of which 70% of the fragmentation products were matched by Mascot, and 40% of the products observed, gave mass differences corresponding to the expected sequence (see Table 6). The entire y-ion series, intriguingly, was shifted by 112.052 Da, suggesting that the y_1 _lysine was modified by an adduct of this mass (see Table 6). A search of the UniMod database of post-translational protein modifications [13] for any modification within 1 Da of this mass, revealed a single entry, acrolein. It is a highly nucleophilic aldehyde that is a product of lipid metabolism, but also forms with thermal decomposition of acrylamide, and was previously shown to form adducts by Michael addition with the ε-amino group of lysine and with cysteine residues [14,15]. However, acrolein has a molecular mass of 56 Da, suggesting that two acrolein adducts formed per lysine residue in the peptide above. Beretta and colleagues previously showed that two such adducts indeed formed on the single lysine residue of the GHK tri-peptide [15].

**Table 6:** Fragment ions of the ^27^KSAPSTGGVK^36 ^peptide from histone H3 isolated from stationary phase *Saccharomyces cerevisiae*.

Order	b ion (*m/z*)	Sequence	y ion (*m/z*)	Order
1	129.1022	K		10
2	216.1343	S	915.4782*	9
3	287.1714	A	828.4462*	8
4	384.2241	P	757.4090*	7
5	471.2562	S	660.3563*	6
6	572.3039*	T	573.3243*	5
7	629.3253	G	472.2766	4
8	686.3468	G	415.2551*	3
9	785.4152	V	358.2336	2
10		K	259.1652	1

The list of product ions observed (underlined), matched with Mascot to the SWISS-PROT database (asterisk superscript), or predicted following CID fragmentation is shown.

Since it is unlikely that trypsin would have cleaved a peptide C-terminal to such a modification, we suggest that the di-acroleination of the C-terminal lysine of KSAPSTGGVK occurred during electrophoresis in the polyacrylamide gel, where acrolein may have been present. We therefore propose that this modification did not occur *in vivo*. Importantly, we found no evidence for acetylation of this lysine residue (K36), unlike the equivalent residue of H3 in exponential phase.

**Figure 7:**
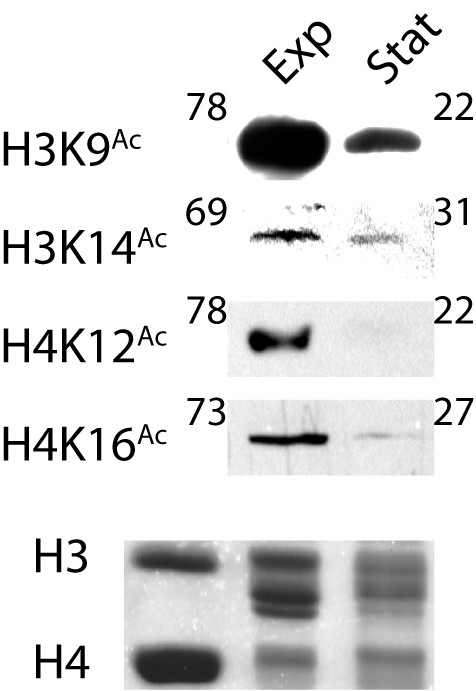
**Western analysis of the acetylation state of histones H3 and H4**. Equal amounts of core histones, isolated from exponential (Exp) and stationary (Stat) phase yeast cells, were resolved by SDS-PAGE, and a Western analysis performed using antibodies directed at acetylated lysine residues H3K9, H3K14, H4K12 and H4K16. A representative Coomassie-stained gel, demonstrating comparable histone H3 and H4 loading, is shown in the bottom panel, with the location of recombinant *Xenopus *H3 and H4 indicated in the left lane. The normalized volume of each band is indicated. The western blot band intensities were normalized by calculating the ratio to the intensity of the corresponding Coomassie-stained histone band, and this ratio was expressed as a percentage of the sum of the ratios for the exponential and stationary phase samples.

To confirm our results and obtain semi-quantitative data on the acetylation levels in exponential and stationary phases, we also performed a Western analysis, using antibodies individually directed at acetylated lysine residues K9 and K14 in H3, as well as K12 and K16 in H4. Histones H3 and H4 isolated from stationary phase yeast cells were acetylated to levels approximately 2 to 3.5 fold lower than that of histones isolated from exponential phase cells (Figure 7).

## Discussion

We have shown above that there was a striking difference in the acetylation level of K9, K14 and K36 of histone H3 and K12 and K16 of histone H4 in exponential phase compared to stationary phase in *S. cerevisiae*. The presence of acetylated lysine residues was determined by a precursor ion scan to detect an *m/z *126 fragment ion, representing the acetylated lysine immonium ion that had lost an ammonia molecule [12]. Hits were verified by a Mascot search of the tandem MS/MS spectra against the SWISS-PROT database, allowing acetylation as a variable modification, as well as by the presence of mass-shifts compatible with an N^ε^-acetyl lysine residue in the y-ion and b-ion fragmentation series. This approach allows a rigorous assessment of the presence of histone acetylation in *S. cerevisiae *cells in exponential and in stationary phase, and can discriminate between the approximately isobaric acetylated and tri-methylated lysine residue.

The acetylation state of the histones are regulated by a balance between acetylation and deactylation activities that are catalyzed by a diverse group of KATs and HDACs, respectively (see Table 7). The target specificity of all KATs and HDAC, which typically form the catalytic sub-unit in multi-component protein complexes, are not yet elucidated, and the precise biological function of acetylation of each of the different lysine residues is also not yet understood. There appears to be a global, background acetylation level that is established by "untargeted" KATs and HDACs, which was proposed to repress basal transcription and also permit the turn-over of acetylation modifications to allow the shutdown of genes were expression were no longer required [16].

**Table 7:** Histone acetyl transferases and deacetylases in *S.cerevisiae*.

Histone	Residue	Acetyl transferase	Deacetylase
	K9	Gcn5 (ScKAT2)	Rpd3
	
**H3**	K14	Gcn5 (ScKAT2) Sas3 (ScKAT6) Sas2 (ScKAT8) Hap2 (ScKAT10)	Rpd3
	
	K36	Gcn5 (ScKAT2)	-

	K5	Hat1 (ScKAT1) Esa1 (ScKAT5)	Rpd3
	
	K8	Hat1 (ScKAT1) Esa1 (ScKAT5)	-
	
**H4**	K12	Hat1 (ScKAT1) Esa1 (ScKAT5)	Rpd3
	
	K16	Esa1 (ScKAT5) Sas2 (ScKAT8)	Rpd3 Sir2

The enzymes responsible for the acetylation and deacetylation of the indicated lysine residues are shown [24].

The acetylation of K5, K8 and K12 of H4 appeared functionally redundant, and only associated with the abolition of the positive charge of lysine. Little difference was seen between the genome-wide transcriptional response of a yeast strain that contained H4 that was mono-acetylated at any of K5, K8 or K12. Furthermore, the effect of acetylation of these residues appeared cumulative, where any of the three di-acetylated combinations had similar, but more pronounced, effects on transcription compared to the mono-acetylated state [17]. In contrast, acetylation of K16 of H4 had specific effects on transcription that were independent of the acetylation state of K5, K8 or K12 [17]. Support for a special, mechanistic role for H4K16 acetylation was also provided by a genome-wide analysis that showed that K16 of H4 in nucleosomes close to transcription start sites were maintained in a hypo-acetylated state, independent of the transcriptional activity of the gene [18]. In contrast, K5 and K12 of H4, as well as K9 and K14 of H3 in nucleosomes located close to transcription start sites, became hyper-acetylated with increase in transcriptional activity [18]. Thus, K16 of H4 appeared less connected with transcriptional regulation than K5 and K12 of H4 and K9 and K14 of H3.

Hansen and co-workers demonstrated that increased acetylation of histones H2B, H3 and H4 decreased the ability of a reconstituted nucleosome fiber to condense *in vitro *[19]. In a subsequent, more targeted study, it was shown that *in vitro *reconstituted oligo-nucleosomes that were fully acetylated at the single K16 of H4 did not condense to the same extent as corresponding un-acetylated fragments [20]. However, both these studies were performed in the absence of linker histone H1, which is required for the formation of a fully condensed 30 nm chromatin fiber [21]. Rhodes and co-workers demonstrated that acetylation of K16 of H4 to a 30% maximal level was sufficient to decondense a 61-mer nucleosomal fiber folded in the presence of a linker histone [22]. Deletion of the H4 tail was also shown to diminish compaction of a reconstituted H1-containing fiber *in vitro *[22], a result similar to that reported for a fiber in the absence of H1 [23]. It was further shown that the region spanning residues 14-19 of H4, encompassing K16, was essential from chromatin compaction in the absence of H1 [23], and, presumably, also in its presence. There is thus an extensive literature that shows that the N-terminal tail of histone H4 was necessary for chromatin compaction, and that acetylation of K16 abolished this compaction.

It was previously shown in *S. cerevisiae *that linker histone Hho1 preferentially bound to chromatin in stationary phase cells, and that this association was a requirement for the formation of condensed chromatin, compared to the decondensed state in exponential phase cells [8]. We therefore propose that the unacetylated state of K16 of H4 reflects a requirement for the absence of this modification to allow the formation of fully condensed chromatin in stationary phase. The presence of acetylated H4K16 in exponential phase is thus likely a reflection of the uncondensed state of the chromatin in active cycling cells, as opposed to the transcriptional status of the genome. A direct correspondence between acetylation level of H3K9, H3K14 and H4K12 and gene expression was previously shown [18]. The acetylation of K9, K14 and K36 of histone H3 and K12 of histone H4 that was observed in exponential phase and the decreased level of acetylation found in stationary phase was therefore most likely modifications that were directly associated with the transcription process.

## Conclusion

We have shown that residues K9, K14 and K36 of histone H3 and K12 and K16 of histone H4 were acetylated at significantly reduced levels in stationary phase compared to exponential phase in yeast. This was shown by both LC/MS/MS and by immuno-blotting in the case of K9, and K14 of histone H3 and K12 and K16 of histone H4. The positive correlation in the data generated by these two techniques confirmed the utility of using the *m/z *126 fragmentation ion as diagnostic of an N^ε^-acetylated lysine residue, allowing unambiguous identification of this modification. The decrease in lysine acetylation in stationary phase most likely reflected the transcriptional shutdown that was previously noted in the semi-quiescent state in yeast [9]. Interestingly, it was previously shown that K16 of H4 was more closely linked to chromatin compaction [22] as opposed to transcriptional activity [18]. We have also previously shown that linker histone Hho1 preferentially bound to chromatin in stationary phase, and was required for chromatin compaction [8]. We therefore suggest that the observed loss of the acetyl group from K16 of H4 is a prerequisite to the full compaction of chromatin in stationary phase.

## Authors' contributions

MN performed the histone isolations, immuno-blots and assisted with the mass spectrometry. GK performed the mass spectrometry. HGP designed the experimental strategy, analyzed the data, prepared the figures and wrote the manuscript. All authors have read and approved the final manuscript.
